# Enhancing the Interfacial Adhesion with Rubber Matrix by Grafting Polydopamine-Carbon Nanotubes onto Poly(p-phenylene terephthalamide) Fibers

**DOI:** 10.3390/polym11081231

**Published:** 2019-07-24

**Authors:** Xuan Yang, Qunzhang Tu, Xinmin Shen, Qin Yin, Ming Pan, Chengming Jiang, Caibing Hu

**Affiliations:** College of Field Engineering, Army Engineering University of PLA, Nanjing 210007, China

**Keywords:** poly(p-phenylene terephthalamide) fibers, dopamine biomimetic modification, aminated carbon nanotubes, interfacial adhesion

## Abstract

To enhance the interfacial adhesion between poly(p-phenylene terephthalamide) (PPTA) fibers and a rubber matrix without damaging the fiber structures, aminated carbon nanotubes (NH_2_-CNTs) were mildly deposited onto the fiber surface by combining the biomimetic modification of dopamine via the Michael addition reaction. Furthermore, differences between the “one-step” method and the “two-step” method were researched through adjusting the addition sequence of NH_2_-CNTs. The surface morphologies and chemical structures of PPTA fibers before and after modification were characterized by scanning electron microscopy (SEM), Fourier transform infrared spectroscopy (FTIR) and X-ray photoelectron spectroscopy (XPS). The mechanical properties of fibers and the adhesive properties with rubber were tested using an electronic tensile tester of single-filament and universal testing machine, respectively. After modification by the “one-step” method for 24 h, the single-filament tensile strength of the modified fibers increased by 16.5%, meanwhile, the pull-out force of the modified fibers to rubber increased by approximately 59.7%. Compared with the “two-step” method, the “one-step” method had superiority due to the short reaction time and the large deposition rate of CNTs.

## 1. Introduction

As a new type of high-performance material, poly(p-phenylene terephthalamide) (PPTA) fibers integrated with high specific strength, outstanding modulus, excellent corrosion resistance and heat resistance are widely used in rubber products to reinforce composites [1,2]. However, the molecular chain of PPTA has high regularity due to the sequential arrangement of the benzene rings and the amide groups at the para sites, and the fibers have a low surface activity, high chemical inertness and weak interfacial adhesion with rubber matrix [3,4]. Thus, the research hotspot now is enhancing the interfacial adhesion between PPTA fibers and rubber matrix via an effective surface modification method.

At present, there some modification methods have been investigated to improve interfacial adhesion of high-performance fibers with a matrix, including ultrasonic [5], high-energy radiation [6,7,8], plasma treatment [9,10], acid etching [11,12], biological enzymes [13,14] and grafting [15,16]. Grafting has development potential that can bring many kinds of nanomaterials and functional groups onto the fiber surface, resulting in the provision of more possibilities for surface modification.

Carbon nanotubes (CNTs), which show extremely high tensile strength of 11–63 GPa and Young’s modulus of 270–950 GPa as well as excellent electrical conductivity and thermal conductivity, have abundant polar groups in their open ends [17,18]. Chen and co-workers [19] first introduced amino groups onto the aramid fibers via a chemical method, and then the acyl chloride-functionalized CNTs were grafted the functionalized surface. The test results showed that the single-filament tensile strength and interlaminar shear strength increased by 12% and 30%, respectively. Similarly, in He and co-workers’ research [20], the aminated CNTs were grafted onto the acyl chloride-functionalized carbon fibers, which can also enhance the interfacial adhesion between fibers and matrix. Rodriguez-Uicab et al. and Ehlert et al. [21,22] grafted acidified CNTs onto the aramid fibers after hydrolysis reaction. However, the test results showed that the single-filament tensile strength of fibers decreased owing to ta severe reaction. Mazeyar Parvinzadeh Gashti and co-workers have also made some interesting progress in the modification of nanomaterials. They prepared the polyvinylpyrrolidone/carbon nanotube/cotton functional nanocomposite with high thermal stability, reduced flammability and good antibacterial properties [23]. They also found that the polypyrrole-MWCNT-Ag composite produced via UV-reduction has higher electrical conductivity and shielding effectiveness compared with the chemically reduced one [24]. Moreover, they researched the effects of the coating of nano- and microemulsion silicones, hydrophilic and hydrophobic nano-silica particles on the thermal properties of composites [25,26]. In our previous work [27], we improved Friedel-Crafts alkylation and then grafted the carboxylated CNTs onto the epoxy functionalized PPTA fibers under the action of an ultrasonic and basic catalyst. This modification method could significantly improve the interfacial adhesion between PPTA fibers and rubber matrix, as well as reduce the mechanical property of fibers slightly. Therefore, on the basis of the previous work, we focus on the selection of the precursor layer in order to effectively graft CNTs without damaging fiber structures.

The mussel adhesive proteins display excellent underwater stickiness, which can be primarily attributed to dopamine (3,4-dihydroxy-phenylalanine) and other catechol compounds [28,29,30]. Dopamine can form a polydopamine (PDA) coating on the surface of almost all kinds of materials under a weak alkaline environment via oxidative self-polymerization reaction. Furthermore, PDA has a high reactivity that can form covalent bonds with thiols and amines through Michael addition or Schiff base reaction [31,32,33]. As a consequence, dopamine is widely utilized in surface modification of high-performance fibers. Sa and co-workers [34] grafted silane coupling agent KH560 onto the PDA precursor layer of aramid fibers and pointed out that the deposition of PDA would not damage the fiber structures. The test results showed that the adhesive strength between modified fibers and rubber increased by 62.5%. In the study of Gong and co-workers [35], the aminated graphene oxide grafted onto the PDA coating of aramid fibers enhanced the interlaminar shear strength between fibers and resin by approximately 34%. Chen et al. [36] reported that the deposited PDA could realize the transformation of carbon fibers from the amphiphobic to the hydrophilic and provide reaction conditions for the subsequent graft of oleophilic groups. As researched by Li and co-workers [37], PDA multilayer films and silane coupling agent KH550 were grafted onto aramid fibers by layer-by-layer assembly. The interlaminar shear strength between modified fibers and resin increased by 45.5%. Dopamine is also commonly utilized to modify nanomaterials. Tian et al. [38] and Zhang et al. [39] deposited PDA onto the surface of nano-silica and CNTs, respectively, followed by grafting amino functional groups via the Michael addition reaction in order to improve the dispersibility and biocompatibilities of nanomaterials. Therefore, we tried to use a PDA coating as the precursor layer of fibers and then grafted aminated CNTs, so as to deposit a large number of CNTs to improve the interfacial adhesion of PPTA fibers with rubber matrix without reducing the tensile strength of fibers.

In this study, we used the “one-step” method and the “two-step” method to modify PPTA fibers, respectively. The former was that the PPTA fibers, dopamine and aminated CNTs were simultaneously put into a weak alkaline aqueous solution (pH = 8.5) for several hours, while the latter was first self-polymerized onto the PPTA fibers and then the aminated CNTs were grafted via the Michael addition reaction. The chemical structures and groups of the PPTA fibers and CNTs were characterized by Fourier transform infrared spectroscopy (FTIR), thermogravimetric analysis (TGA), and X-ray photoelectron spectroscopy (XPS). The morphologies of the fibers were observed by scanning electron microscope (SEM). The fiber strength and the adhesive properties between fibers and rubber were tested by electronic tensile tester of single-filament and universal testing machine, respectively.

## 2. Materials and Methods

### 2.1. Materials

PPTA fibers, Kevlar^®^ K-29 (dtex 3300) with an average diameter of 14 μm, were purchased from Changzhou Gaoyuan Group Co., Ltd., Changzhou, China. Aminated single-walled CNTs (NH_2_-CNTs) with a mean diameter of 8–15 nm, a length of 5 μm and an amino content of 0.45 wt.%, and untreated single-walled CNTs (A-CNTs) with a mean diameter of 8–15 nm and length of 0.5–2 μm were both purchased from Nanjing XFNANO Materials Tech Co., Ltd., Nanjing, China. Dopamine hydrochloride, tris (hydroxymethyl) aminomethane (Tris), ethyl acetate and acetone were of analytical reagent grade and purchased from Aladdin Industrial Co., Ltd., Shanghai, China. The rubber ingredients, as displayed in Table 1, were of industrial grade and purchased from Nanjing Xinyue Chemical Industrial Co., Ltd., Nanjing, China.

### 2.2. The “One-Step” Method of Surface Modification of PPTA Fibers

PPTA fibers were first cleaned with acetone and ethyl acetate by turns, followed by drying in a vacuum oven at 80 °C for 3 h. The dried fibers were labeled as A-PPTA (as-received PPTA). NH_2_-CNTs (150 mg) and 50 mg A-PPTA were first added into 150 mL dopamine hydrochloride solution with a concentration of 2 g/L (pH = 8.5), and then the suspension was stirred slowly at room temperature for 12 h, 18 h and 24 h, respectively. After that, the modified fibers were washed several times with deionized water, followed by drying in a vacuum oven at 80 °C for 3 h. According to the reaction time, the dried fibers were labeled as PPTA-1, PPTA-2 and PPTA-3, respectively.

In the controlled experiment, NH_2_-CNTs were replaced by A-CNTs and the reaction time was 24 h. The cleaned and dried fibers were labeled as PPTA-4.

### 2.3. The “Two-Step” Method of Surface Modification of PPTA Fibers

A-PPTA fibers (50 mg) were first added into 150 mL dopamine hydrochloride solution with a concentration of 2 g/L (pH = 8.5) and then stirred slowly at room temperature for 12 h. The fibers after the reaction were washed several times with deionized water, followed by drying in a vacuum oven at 80 °C for 3 h. The dried fibers were labeled as PDA-PPTA. PDA-PPTA fibers were immersed into 150 mL NH_2_-CNTs aqueous suspension with a concentration of 1 g/L (pH = 8.5) under slow stirring at room temperature for 24 h. The fibers after modification were cleaned and dried in accordance with the mentioned process, and then were labeled as PPTA-5.

**Figure 1:**
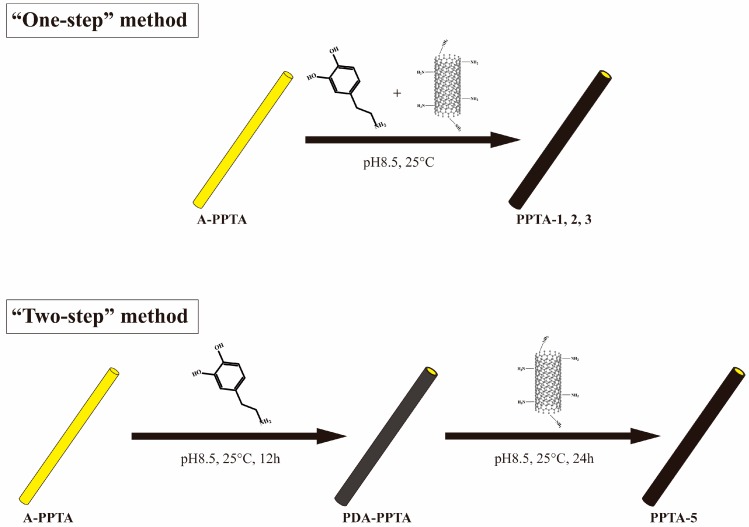
Schematic diagrams of surface modification.

The schematic diagrams of the “one-step” method and the “two-step” method are presented in Figure 1.

### 2.4. Preparation of PPTA Fibers/Rubber Composites

**Figure 2:**
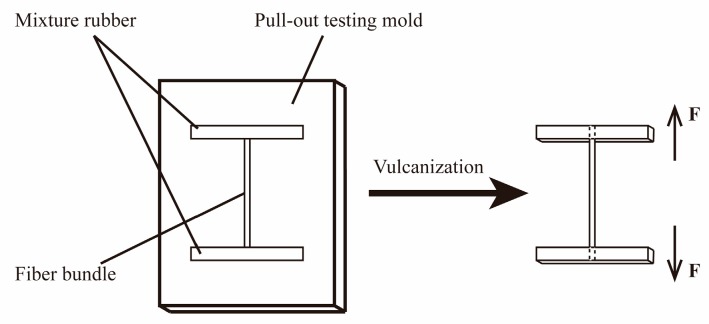
The schematic diagram of the pull-out force test.

PPTA fibers/rubber composites were prepared for pull-out tests to measure the adhesive properties between PPTA fibers before and after modification and rubber matrix. The rubber ingredients, listed in Table 1, were first mixed in an internal mixer at 80 °C for 10 min. Afterwards, the rubber mixture was cut into some small sheets, followed by placing into the pull-out testing mold. PPTA fiber bundles were then embedded onto the sheets and covered with another sheet. Finally, the mold was closed and vulcanized at 140 °C for 45 min under 20 MPa. The schematic diagram of the pull-out force test can be seen in Figure 2. Where “F” represents the direction of the machine force.

### 2.5. Characterizations

SEM (FEI Nova NanoSEM 230, Hillsboro, OR, USA) was utilized to observe the surface morphologies of PPTA fibers before and after modification. FTIR (PerkinElmer Spectrum Two, Waltham, MA, USA) was performed to characterize the chemical groups of PPTA fibers and CNTs. TGA (TG 209 F3 Tarsus, Selb, Germany) was carried out to obtain the thermal decomposition process in the temperature range of 30 °C–800 °C with a heating rate of 10 °C/min under a nitrogen (N_2_) atmosphere. The wavenumber range was 4000–650 cm^−1^. The surface chemical compositions of fibers were characterized by XPS (ESCALAB 250XI, Thermo Electron Corporation, Waltham, MA, USA). The C 1s line at 284.6 eV was taken as the reference of all binding energies to compensate for the surface charging effects and XPS Peak 4.1 was utilized to fit peaks in high-resolution spectra. According to the standard ISO 11566-1996, electronica tensile tester of single-filament (YM-06B, Shaoxing, China) was carried out to test the tensile strength of PPTA fibers before and after modification. The measuring range of the load cell (EVT-10H, Shanghai Yu Ran Sensor Technology Co., Ltd., Shanghai, China) was 0–50 N. The gauge length and tensile rate were 20 mm and 5 mm/min, respectively. The average values of the measured results were obtained from at least 20 specimens. According to the standard GB/T 2942-2009, universal testing machine (ETM104C, Wance Testing Machine, Shenzhen, China) was carried out to test the pull-out force and the tensile rate was 100 mm/min. The measuring range of the load cell (YZC-522, Shenzhen Youzhongli Technology Co., Ltd., Shenzhen, China) was 0–1000 N. The average values of the measured results were obtained from at least eight specimens.

## 3. Results and Discussion

### 3.1. Surface Morphologies of PPTA Fibers

**Figure 3:**
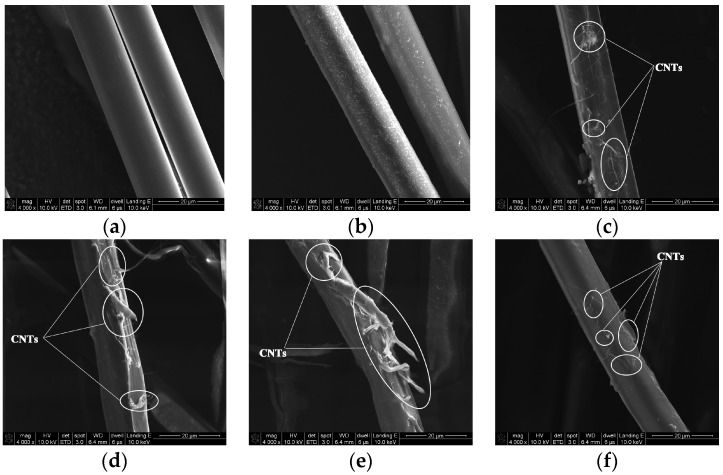

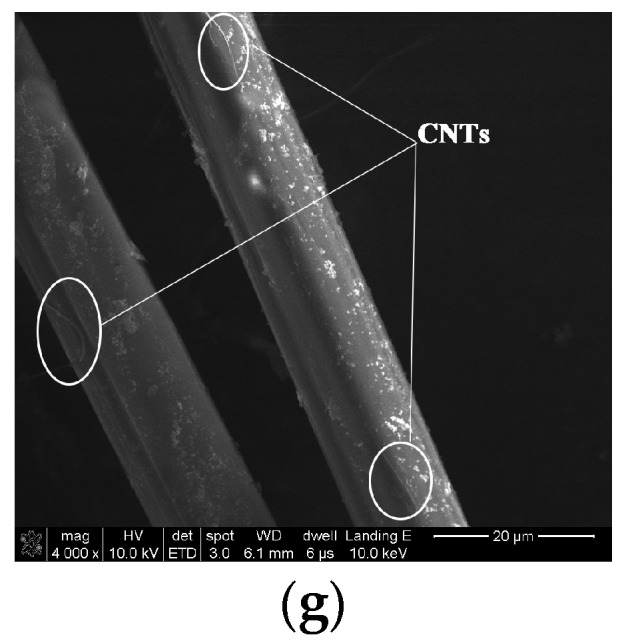
Surface morphologies of Poly(p-phenylene terephthalamide) (PPTA) fibers: (**a**) A-PPTA; (**b**) PPTA-PDA; (**c**) PPTA-1; (**d**) PPTA-2; (**e**) PPTA-3; (**f**) PPTA-4; (**g**) PPTA-5.

The surface morphologies of PPTA fibers under different modification conditions can be clearly observed by SEM, which can provide the basis for the subsequent analyses. As shown in Figure 3a, the surface of A-PPTA was smooth and clean. Compared with Figure 3a, it can be seen from Figure 3b that the fiber surface had obviously deposited PDA particles. Figure 3c,e shows the surface morphologies of PPTA-1, PPTA-2 and PPTA-3, respectively. It can be observed from these that with the increase of reaction time, the amount of CNTs increased apparently. Figure 3f shows the surface morphology of PPTA-4 fibers, which were grafted A-CNTs through the “one-step” method for 24 h. As can be seen from this figure, the amount of CNTs was as small as that in Figure 3c, indicating that NH_2_-CNTs can promote the deposition onto the fiber surface. With the short reaction time, the biomimetic modification of dopamine on the fiber surface can play the role as a bridge, namely, dopamine was capable of absorbing CNTs in suspension while polymerizing onto the surface. With the long reaction time, PDA formed on the fiber surface can further react with NH_2_-CNTs via the Michael addition reaction, increasing the number of CNTs on the surface. On account of the absence of functional groups, such as amino groups, the deposition of A-CNTs on the fiber surface was less. In comparison with Figure 3b, a small number of CNTs can be observed on the surface of PPTA-5 which were prepared by the “two-step” method as shown in Figure 3g, which were just deposited through the Michael addition reaction.

### 3.2. Chemical Structures of PPTA Fibers

**Figure 4:**
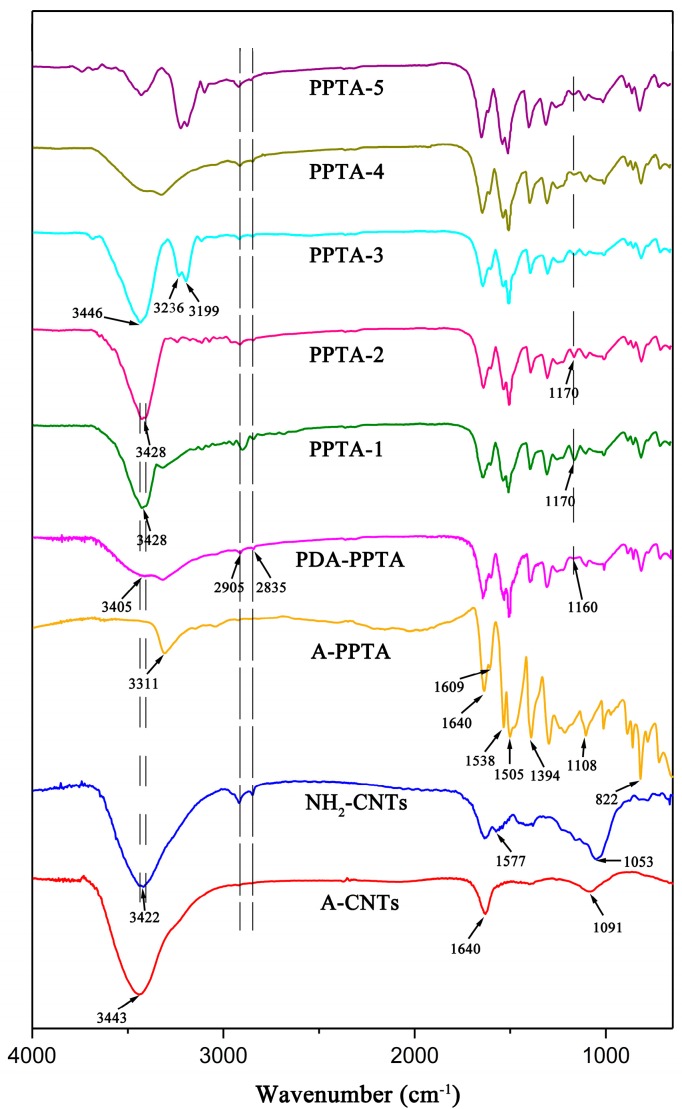
FTIR spectra of CNTs and PPTA fibers.

In the reaction mechanism of the “one-step” method, dopamine can play the role of a bridge to adsorb an amount of NH_2_-CNTs and wrap them onto the fiber surface with oxidative self-polymerization reaction, and then, with the increase of reaction time, more NH_2_-CNTs can be absorbed onto PDA film by subsequent Michael addition reactions. In the reaction mechanism of the “two-step” method, NH_2_-CNTs can be deposited onto the fiber surface only by the Michael addition reaction due to the formation of a PDA precursor layer. For the purpose of the analyses of the surface modification process, the chemical structures of CNTs and fibers were characterized by FTIR as illustrated in Figure 4.

Atmospheric moisture or oxidation during the purification of A-CNTs may lead to the generation of -OH and C-O, whose stretching vibration would respectively result in the peaks at 3443 cm^−1^ and 1091 cm^−1^ as shown in A-CNTs spectrum [40]. Another peak at 1640 cm^−1^ is attributed to the stretching vibration of C=O in quinone groups [41]. In NH_2_-CNTs spectrum, a sharp doublet can be observed near 3422 cm^−1^, which is related to the stretching vibration of N-H in primary amine groups. Meanwhile, the peaks at 1577 cm^−1^ and 1053 cm^−1^ result from the bending vibration of N-H and the stretching vibration of C-N, respectively.

In the spectrum of A-PPTA, the stretching vibration and the bending vibration of N-H in amide groups generate the peaks at 3311 cm^−1^ and 1538 cm^−1^, respectively. Besides, the peaks at 1640 cm^−1^ and 822 cm^−1^ are attributed to the stretching vibration of C=O and the bending vibration of C-H, respectively. Meanwhile, the two peaks at 1394 cm^−1^ and 1108 cm^−1^ result from the stretching vibration of C-N and the two peaks at 1609 cm^−1^ and 1505 cm^−1^ are related to the benzene rings [42,43]. In the spectrum of PDA-PPTA, the new peaks at 3405 cm^−1^ and 1160 cm^−1^ can be ascribed to the stretching vibration of -OH and C-O, respectively. Furthermore, the stretching vibration of methylene results in the new double peaks at 2905 cm^−1^ and 2835 cm^−1^. In the spectra of PPTA-1 and PPTA-2, there are new doublets near 3428 cm^−1^, which can be attributed to the stretching vibration of N-H in primary amine groups. Meanwhile, the absorption peak at 1170 cm^−1^ generated from the stretching vibration of C-O becomes sharper. Comparatively, in the spectrum of PPTA-3, the doublets near 3428 cm^−1^ are replaced by a singlet at 3448 cm^−1^; meanwhile, the new double peaks appear at 3236 cm^−1^ and 3199 cm^−1^. The changes of absorption peaks can be ascribed to the stretching vibration of N-H in aromatic secondary amine groups and C=N, respectively, which can confirm the proceeding of the Michael addition reaction. In the spectrum of PPTA-4, no new peaks appeared compared with that of PDA-PPTA, indicating that the deposition of A-CNTs onto the PPTA fibers does not generate new groups. The spectrum of PPTA-5 is similar to that of PPTA-3, illustrating that NH_2_-CNTs are finally deposited onto the fiber surface via the Michael addition reaction in the “two-step” method. From the FTIR spectra and above analyses, it can be obtained that in the “one-step” method, NH_2_-CNTs would be deposited onto the fiber surface via oxidative self-polymerization of dopamine with the short reaction time. While the reaction time increases, more NH_2_-CNTs would be further deposited onto the fiber surface through the subsequent Michael addition reactions.

**Figure 5:**
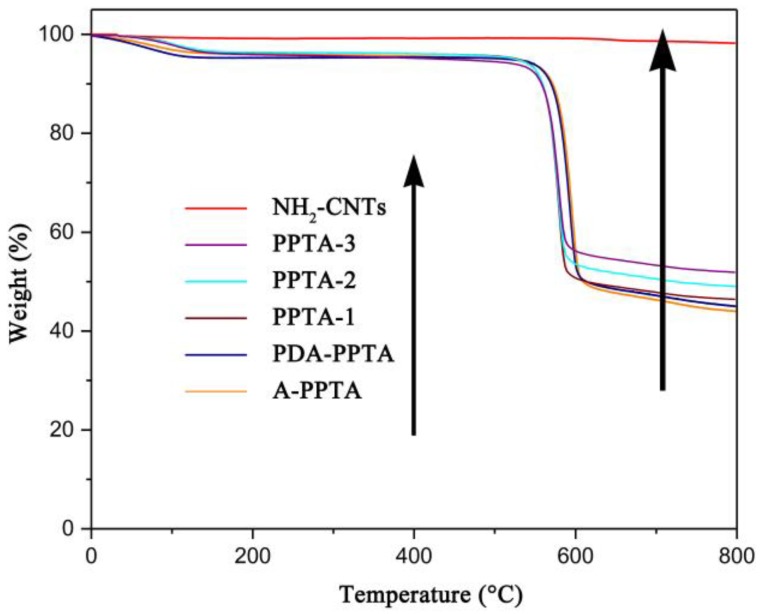
TGA curves of PPTA fibers and NH_2_-CNTs in the range of 30 °C to 800 °C.

TGA tests in a flowing N_2_ atmosphere were utilized to obtain the thermal decomposition process of the fibers. Figure 5 presents the TGA curves of NH_2_-CNTs and PPTA fibers before and after modification. The TGA curve of NH_2_-CNTs shows a slight weight loss of 1.76% in the temperature range of 30 °C to 800 °C. The TGA curve of A-PPTA shows an obvious weight loss of 57.38%, which can be mainly related to the decomposition of partial dehydroxylation and alkoxide of PPTA fibers [44]. Compared with it, the weight loss in the range of 30 °C to 800 °C of PDA-PPTA fibers can be obtained to be 56.92% in the TGA curve. The decrease can be attributed to the deposition of PDA that has a lower thermal weight loss (about 50%) [36]. After modification by the “one-step” method, the weight loss in the range of 30 °C to 800 °C for PPTA-1, PPTA-2 and PPTA-3 fibers was 56.24%, 55.23% and 54.21%, respectively, indicating that the gradual increase of CNTs with the increase of reaction time.

**Figure 6:**
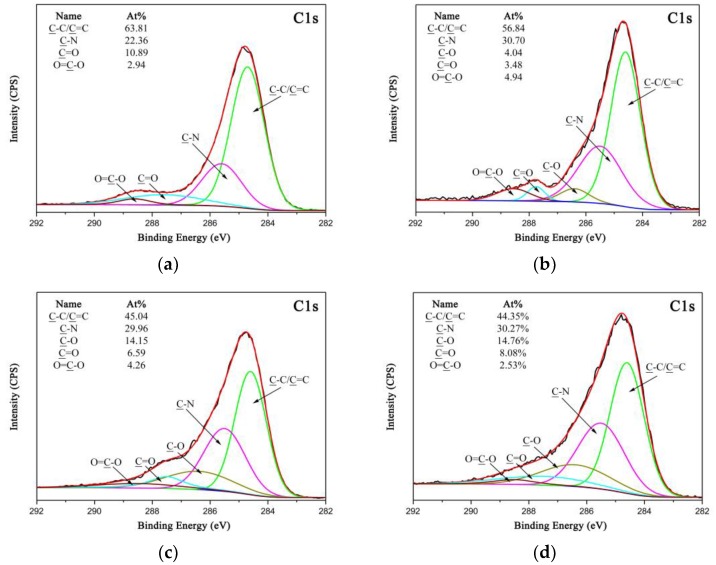
High-resolution C 1s XPS spectra of PPTA surface: (**a**) A-PPTA; (**b**) PPTA-3; (**c**) PPTA-4; (**d**) PPTA-5.

The chemical compositions of the PPTA fibers before and after modification can be characterized by XPS. In Figure 6a, the high-resolution C 1s XPS spectrum of A-PPTA can be fitted with four peaks, which are attributable to C-C/C=C at 284.6 eV, C-N at 285.5 eV, C=O at 287.5 e V and O=C-O at 288.5 eV, respectively [45]. In the high-resolution C 1s XPS spectra of PPTA-3 and PPTA-4 as respectively demonstrated in Figure 6b,c, the newly fitted peak at 286.4 eV can be related to C-O in phenolic hydroxyl groups of PDA. Moreover, the concentrations of C-N of PPTA surface dramatically increase from 22.36 at.% to 30.70 at.% and 29.96 at.% respectively, which can also confirm the proceeding of dopamine oxidative self-polymerization. Comparing Figure 6b and Figure 6c, it can be observed that the concentration of C-C/C=C of PPTA-3 surface after grafting NH_2_-CNTs is much higher than that of PPTA-4 surface after grafting A-CNTs, which can be attributed to the synergistic action of oxidative self-polymerization and the Michael addition reaction resulting in a higher deposition amount of CNTs. As shown in Figure 6d, the concentrations of fitted chemical groups of PPTA-5 surface are similar to that of PPTA-4. In combination with the low amino content of NH_2_-CNTs, it can be obtained from the results of SEM, FTIR and XPS that the deposition of NH_2_-CNTs has a negligible effect on the concentration of C-N on the fiber surface.

### 3.3. The Single-Filament Tensile Strength of PPTA Fibers

**Figure 7:**
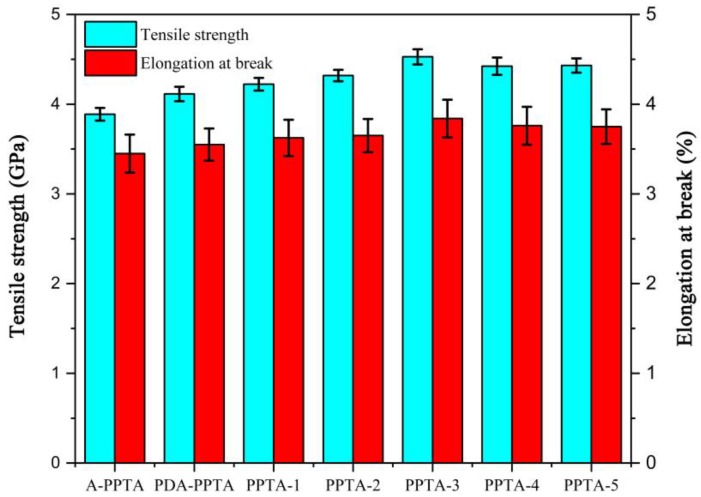
The mean values of single-filament tensile strength and elongation at break of PPTA fibers.

In our previous work [27], the fiber structures would be damaged due to the intensity of the Friedel-Crafts alkylation, and the fiber strength reduced slightly [46]. It can be known that the formation of the PDA precursor layer on the PPTA fiber surface may be ascribed to the conjugate action of the conjugated π bond electron cloud of benzene rings and the coating effect of dopamine self-polymerization [45,47], which would not damage the fiber structures theoretically. In order to verify the mildness of the surface modification, the single-filament mechanical properties of PPTA fibers before and after modification were tested. Figure 7 presents the results of tensile strength and elongation at break.

The single-filament tensile strength (GPa) of PPTA fibers was 3.89 ± 0.07, 4.11 ± 0.08, 4.22 ± 0.07, 4.32 ± 0.06, 4.53 ± 0.08, 4.42 ± 0.10 and 4.43 ± 0.08, respectively. The elongation at break (%) was 3.45 ± 0.21, 3.55 ± 0.18, 3.63 ± 0.20, 3.65 ± 0.18, 3.84 ± 0.21, 3.76 ± 0.21 and 3.75 ± 0.19, respectively. As demonstrated in Figure 7, the strength of PDA-PPTA fibers has been improved after the biomimetic modification of dopamine. It can be illustrated that the coating effect of dopamine self-polymerization weakens the stress concentration of fibers and plays a positive role in the reinforcement of fibers. The single-filament tensile strength of PPTA-1, PPTA-2 and PPTA-3 increases gradually and the highest strength has increased by 16.5%, indicating that with the increase of reaction time of the “one-step” method, the mechanical properties of fibers would be enhanced greatly by the increasing CNTs. Compared with the increased amplitude of the single-filament tensile strength of PPTA-3, that of PPTA-4 and PPTA-5 was not apparent. This is owing to that the tensile stress sharing generated by low deposition of CNTs on PPTA-4 surface and PPTA-5 surface was not as good as that on PPTA-3 surface. Meanwhile, with the enhancement of tensile strength, the maximum elongation at break increased by 11.3%, which can also illustrate that the mechanical properties of fibers has been improved after the deposition of NH_2_-CNTs via the “one-step” method.

### 3.4. The Adhesive Property between PPTA Fibers and Rubber

**Figure 8:**
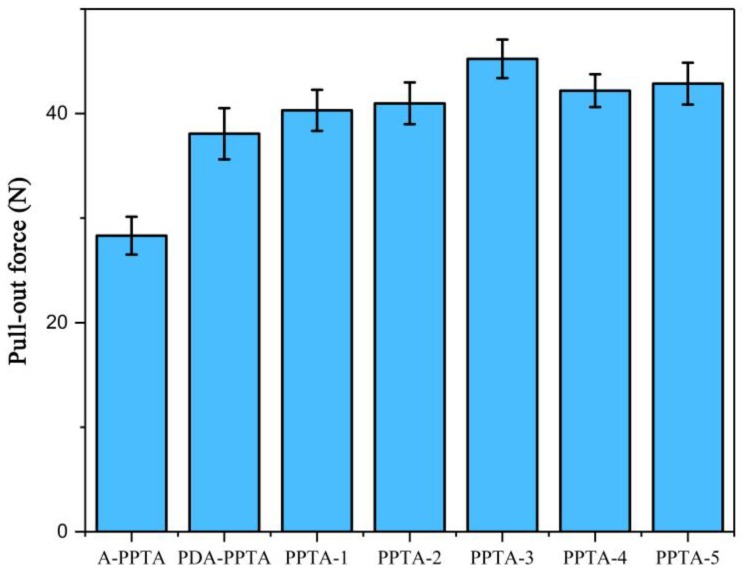
The pull-out results of PPTA fibers with rubber.

The results of the pull-out tests between PPTA fibers and rubber are shown in Figure 8, which can be utilized to characterize the interfacial properties of PPTA fibers reinforced rubber composites. Compared with the pull-out force of A-PPTA fibers to rubber, that of PDA-PPTA fibers to rubber increased by approximately 34.6% (from 28.3 N to 38.1 N), which indicates the deposition of PDA can improve the surface activity of the fibers. It can also be obtained from Figure 8 that PPTA-3 has the highest pull-out force that increased by approximately 59.7% compared with that of A-PPTA (from 28.3 N to 45.2 N).

**Figure 9:**
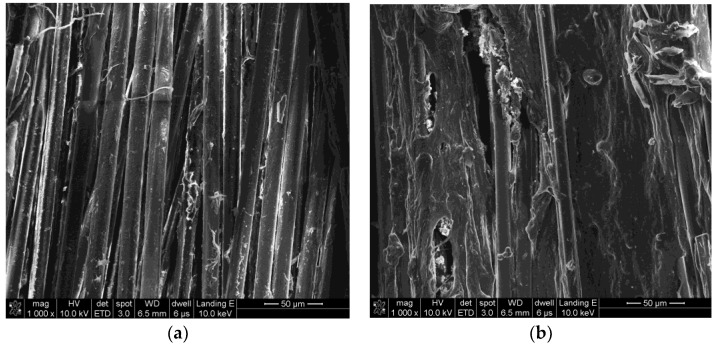

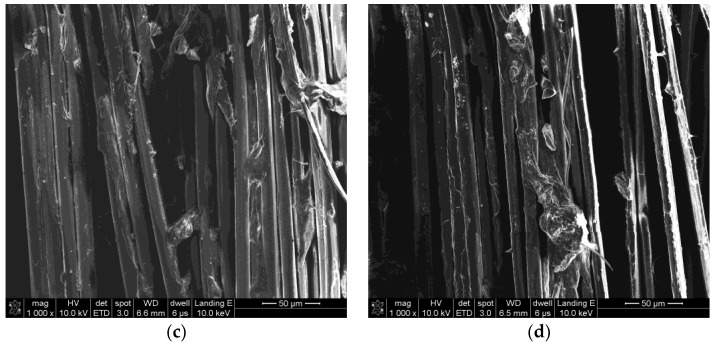
The surface morphologies of PPTA fibers after pull-out: (**a**) A-PPTA; (**b**) PPTA-3; (**c**) PPTA-4; (**d**) PPTA-5.

Figure 9 shows the surface morphologies of PPTA fibers after the pull-out tests. In Figure 9a, there is little residual rubber remaining on the A-PPTA fiber surface, indicating the surface of the A-PPTA fiber was so smooth that the interaction between fibers and rubber was weak. Comparatively, it can be seen from Figure 9b that a large amount of residual rubber remained on the PPTA-3 fiber surface and the fracture point was located at the rubber matrix. The amount of residual rubber in Figure 9c is similar to that in Figure 9d, besides, the fracture points were located near the interface of the composites. Furthermore, the crosslinking between fibers had been generated.

The adhesive properties between fibers and rubber were enhanced remarkably via the “one-step” method modification for 24 h, and the reasons can be illustrated by the following two aspects. Firstly, the deposition of NH_2_-CNTs can enhance the roughness of the fiber surface as well as form interlaced network structures to generate mechanical interlocking with rubber matrix [48]. Secondly, the polar groups in the open ends of NH_2_-CNTs may promote the surface energy of the fibers, which would improve the affinity between PPTA fibers and the rubber matrix.

## 4. Conclusions

In this work, NH_2_-CNTs were successfully deposited onto the surface of PPTA fibers by combining the biomimetic modification of dopamine with the Michael addition reaction, which effectively improved the interfacial adhesion between PPTA fibers and rubber the matrix. SEM, FTIR and XPS were carried out to study the modification process of the fibers. The electronic tensile testing of a single-filament and a universal testing machine were performed to test the mechanical properties of the fibers and the adhesive properties with rubber, respectively. The results showed that the PPTA fibers modified by the “one-step” method for 24 h had grafted the largest amount of CNTs. At this point, the single-filament tensile strength of modified fibers increased by 16.5%. Moreover, the pull-out force of modified PPTA fibers to rubber increased by approximately 59.7%. Comparatively, the “two-step” method lacked superiority due to the long reaction time, the low deposition rate of CNTs, the slight enhancement of mechanical and adhesive properties. The surface modification method proposed in this study, which could improve the interfacial adhesion between PPTA fibers and rubber with enhancing the fiber strength, is mild and effective. It could be predicted that this novel method would be applied to the study of more kinds of polymer modification.

## Figures and Tables

**Table 1:** Rubber formulation.

Materials	Parts per Hundreds of Rubber
Styrene-butadiene rubber	60
Natural rubber	40
Antioxidant (4010NA)	1.5
Carbon black	20
White carbon black	15
Zinc oxide	5
Stearic acid	2.5
Aromatic oil	10
Coumarone indene resin	10
Rubber adhesive (RA)	1
Rubber adhesive (RS)	1
Accelerant (CZ)	5
Sulphur	1
Total	172
